# Supplementary material for: Non-Inflamed Tumor Microenvironment and Methylation/Downregulation of Antigen-Presenting Machineries in Cholangiocarcinoma
Source: Cancers (Basel). 2023 Apr 20;15(8):2379. doi: 10.3390/cancers15082379 (PMC10136850; doi:10.3390/cancers15082379)
Supplement: Supplementary file 1 [file cancers-15-02379-s001.zip › cancers-2301470-supplementary.pdf]

Supplementary figures

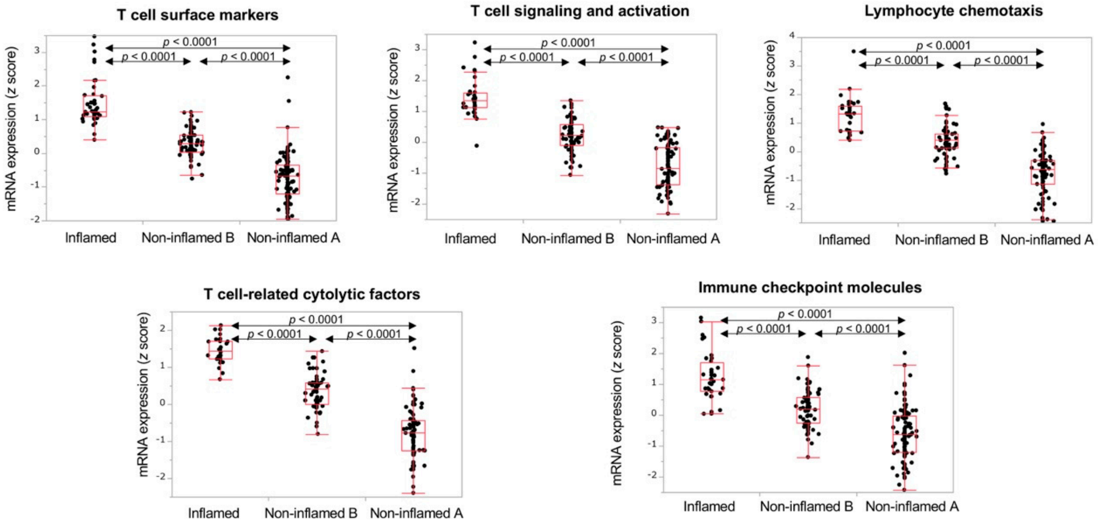

Figure S1: Comparisons of mRNA expression of TIL-related markers among inflamed, non-inflamed A, and non-inflamed B subgroups.

The following genes were selected as TIL-related markers; *CD2*, *CD3D*, *CD8A*, *CD8B*, *CD48*, *CD52,* and *CD53* as T cell surface markers; *FYB1*, *IFNG*, *LAPTM5*, *LCP2*, *PTPRC,* and *SLA* as T cell signaling and activation-related markers; *CCL4*, *CCL5*, *CXCL9*, *CXCL10*, *CXCL11,* and *CXCR4* as lymphocyte chemotactic markers; *GZMA*, *GZMB*, *GZMK*, *GZMH,* and *GZMM* as T cell-related cytolytic factors; *CTLA4*, *LAG3*, *TIGIT*, *CD274*, *PDCD1,* and *HAVCR4* as immune checkpoint molecules. *Z*-scores of the mRNA levels are applied for normalization among multiple genes in T cell surface markers, T cell signaling and activation-related markers, lymphocyte chemotactic markers, T cell-related cytolytic factors, and immune checkpoint molecules. Red boxes and whiskers plots denote 75% and 95% distribution, respectively, and the red lines in the boxes show the median values. *P*-values for multiple comparisons by non-parametric Steel-Dwass' test are shown.

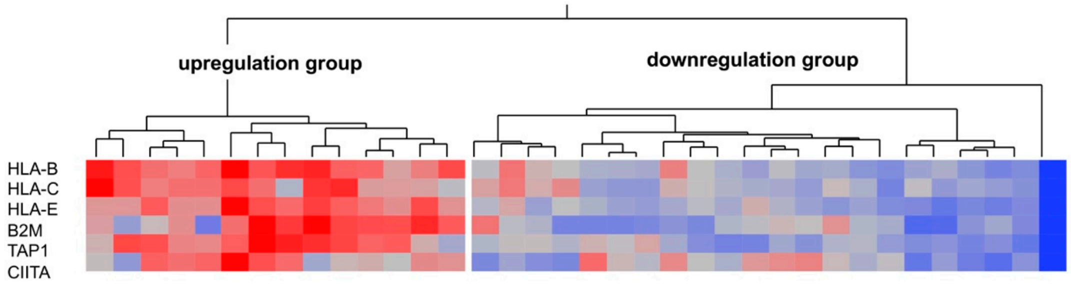

Figure S2: Classification of cholangiocarcinoma with hierarchical clustering analysis using mRNA levels of antigen-presenting machinery.

We performed hierarchal clustering analysis to classify tumors based on gene expression involved in antigen presentation. Of the 36 cholangiocarcinomas, 14 (38.9%) were classified into the "upregulated" and 22 (61.1%) into the "downregulated" expression groups, respectively. The genes involved in the antigen-presenting machinery include *HLA-B, HLA-C, HLA-E, B2M, TAP-1,* and *CIITA*, which are previously applied for the classification of HCC cases (Montironi C, Castet F, Haber PK, Pinyol R, Torres-Martin M, Torrens L, et al. Inflamed and non-inflamed classes of HCC: a revised immunogenomic classification. Gut 2023;72:129-140).

(a)

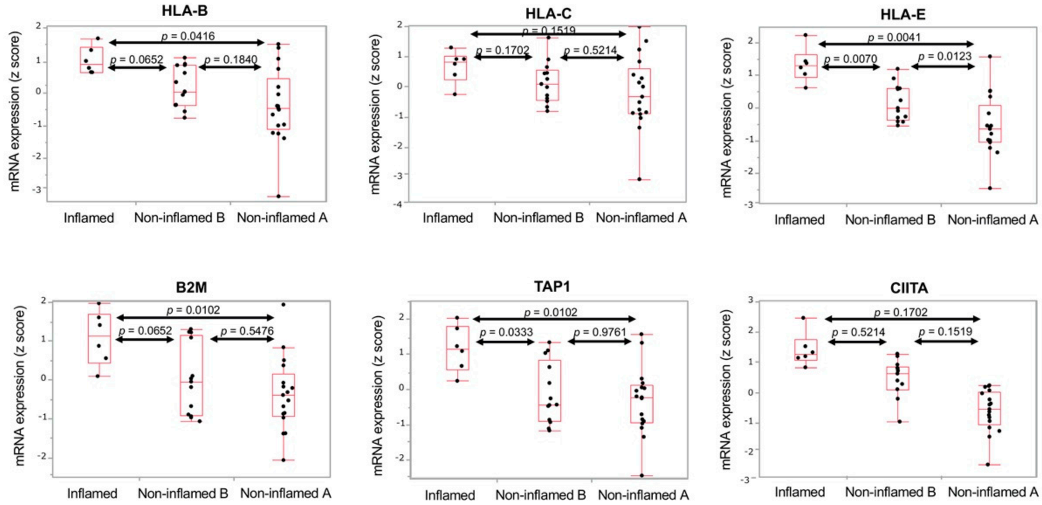

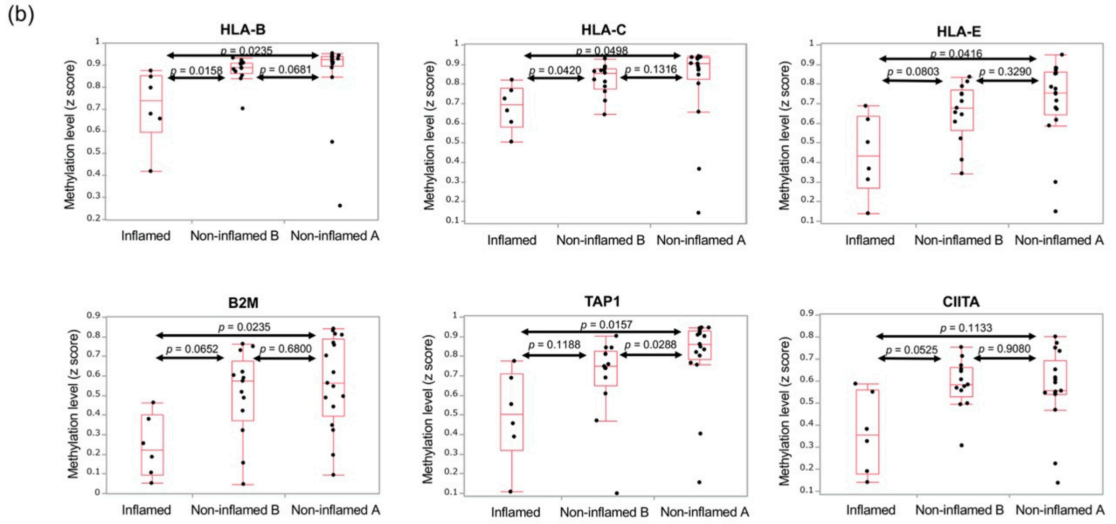

Figure S3: Comparisons of mRNA expression and DNA methylation level of antigen-presenting machineries among inflamed, non-inflamed A, and non-inflamed B groups.

The inflamed subtype shows the highest mRNA expression (a) and the lowest DNA methylation level among three subtypes (b). For the comparison between non-inflamed A and non-inflamed B subgroups, median mRNA expression levels are lower in tumors in non-inflamed A than those in non-inflamed B for all 6 antigen-presenting machineries but TAP1 (a). Similarly, median DNA methylation levels are higher in tumors in non-inflamed A than those in non-inflamed B in all 6 antigen-presenting machineries but B2M and CIITA (b).

Red boxes and whiskers plots denote 75% and 95% distribution, respectively, and the red lines in the boxes show the median values. *P*-values for multiple comparisons by non-parametric Steel-Dwass' test are shown.

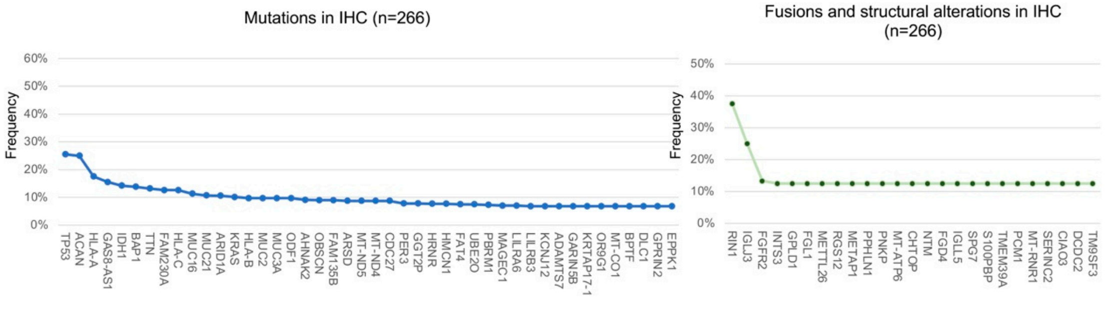

Figure S4: Genes recurrently altered in cholangiocarcinoma.

Genomic data from 1,576 cholangiocarcinoma samples from eleven cohorts were deposited in the cBioPortal public database (https://www.cbioportal.org). Among them, status of gene mutations, and fusions and structural alterations are available for 266 cholangiocarcinomas. Frequencies of gene mutations (left panel), and fusions/structural alterations (right panel) are shown by descending order.

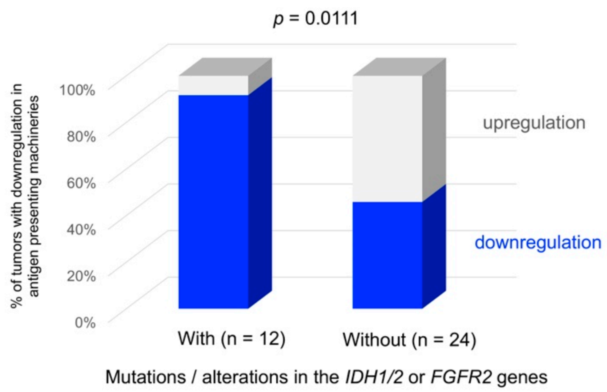

Figures S5: Comparison of frequencies of cholangiocarcinomas with downregulation of antigen-presenting machineries between tumors with and without mutations/alterations of the IDH1/2 or *FGFR2* genes

Frequencies of cholangiocarcinomas with downregulation of antigen-presenting machineries are shown in tumors with and without mutations/alterations of the IDH1/2 or *FGFR2* genes. Downregulation of antigen-presenting machineries is significantly more frequent in the tumor with mutations/alterations of the *IDH1/2* or *FGFR2* genes than those without (p = 0.0111).
